# Comparative Analysis of Factors Affecting Quality of Community-Based Care Services in Korea

**DOI:** 10.3390/ijerph19084641

**Published:** 2022-04-12

**Authors:** Kichan Yoon, Munjae Lee

**Affiliations:** 1 School of Health Policy & Management, Health Science College, Korea University, Seoul 02841, Korea; ykichan08@gmail.com; 2 Department of Medical Humanities and Social Medicine, School of Medicine, Ajou University, Suwon 16499, Korea; 3 Medical Research Collaborating Center, Ajou Research Institute for Innovative Medicine, Ajou University Medical Center, Suwon 16499, Korea

**Keywords:** social service, care service, community care, user satisfaction, Korea

## Abstract

Aging societies have an increased need for care services. To solve the problem of care, we suggest community care, through which medical services are provided that meet individual needs. Korea provides care services in advance of the community care project and implements quality control to improve the quality of these social services. Therefore, this study aims to compare and analyze the factors affecting user satisfaction in care services in both 2013 and 2016. We analyzed secondary data from 2013 and 2016 collected by the Social Security Information Service. These data include standardized metrics for the quality of care. Based on the evaluation indexes for care service in 2013 and 2016, we used commonly used indexes for analysis. Specifically, non-profit organizations were influenced by sales, accounting management, lifetime tenure rate, etc., while for-profit organizations were affected by number of users, contract termination, etc. In addition, on-site evaluation had a negative effect on the change rate of user satisfaction. Therefore, in order to increase satisfaction with care services, evaluation indexes by service type should be diversified and differentiated. In addition, field evaluations related to user satisfaction should be performed in order to provide care services appropriate for local characteristics.

## 1. Introduction

By August 2017, Korea had already become an “aged society,” in which the proportion of older adults aged 65 or older exceeded 14%. By 2026, the older adults population will thus exceed 10 million, and dementia patients will account for about 10% (1 million people) of the total older adults population; this implies that issues related to the health care and welfare of the older adults will be a serious social problem [1,2,3]. The advent of such an aged society will lead to a rapid increase in the need for care services for older adults and in the care burden for their families. To address these care-related problems, the government announced the Master Plan for Community-Integrated Care in November 2018; since June 2019, 16 municipalities have been participating in the pilot project [4].

In Korea, most of the social services use facility-oriented care services targeting older adults, people with disabilities, children, etc. As of the end of 2016, a total of 4331 facilities, including mental health care facilities, facilities for the handicapped, older adult care facilities, child care facilities, and facilities for the homeless, were in operation; the number of residents had reached 189,782 people [5]. However, these kinds of institutional care have increasingly come to be regarded negatively due to residents’ loss of identity; restrictions on basic freedoms; their hierarchical structure, which makes it difficult to achieve rehabilitation goals; a style of communal living that is routinized and centered on regulations; and the psychological distance between residents and employees, among other things [6,7,8]. As an alternative to these types of institutional care, de-institutionalization has been suggested; this involves former residents leading a self-reliant, autonomous life in the community, instead of living in a dependent state in large institutions [9,10].

As part of such de-institutionalization policies, community care has been suggested [11,12,13]. Through community care, recipients in need of care are provided with the benefits of welfare and medical services that meet their needs while they live in the community, in their own houses, group homes, or the like. Community care is also a system through which self-realization and daily activities are enabled as part of the community [14,15,16,17]. Before the full community care project began, the government, through a pilot project initiated in 2010, began providing three major at-home care services for postpartum women and infants, house and health help, and older adult care in 2012.

The pilot project was conducted specifically for a QE (Quality Evaluation) of social services, targeting 143 organizations providing care services for postpartum women and infants in 2010 and 319 organizations providing home and health help/older adult care services in 2011. Subsequently, social services quality control work was initiated to protect social service users and to evaluate the quality of social services offered by providers in accordance with the quality criteria of social services under Article 30 of the Act on the Use of Social Services and the Management of Social Service Vouchers, which has been enforced since 2012 [18,19].

In Korea, care services are provided by a public institution called the Social Security Information Service (SSIS), with the nation providing financial support. Sweden provides a comparable system, where the quality control of services for the general public is undertaken by the Inspektionen for vard och omsorg (IVO), which was established for the quality control, research, and oversight of the health and welfare services. Sweden’s IVO, like the SSIS in South Korea, provides evaluation criteria for the social services, conducts assessments for quality control, and applies measures for evaluating results [20,21,22]. Both SSIS in Korea and IVO in Sweden have a common point in that they evaluate the government-led care projects, but in Korea, quality evaluation is the main purpose, and the follow-up management is limited, as it does not have any authorities for investigation or supervision similar to what the Sweden’s IVO has.

Nevertheless, a detailed analysis has not been conducted on care services, the core project of community care that was executed in both 2013 and 2016. In particular, to ensure that the community care project is successful in the future, it may be more important than anything else to identify the factors influencing care services and to give direction accordingly [23,24]. Furthermore, it may be noted that the current evaluation method of care services is implemented without any distinction between service type and operational bodies (for-profit vs. non-profit) [13,25,26,27].

Therefore, because care services are implemented by the SSIS in Korea, in this study we intend to compare and analyze the factors affecting user satisfaction in care services in both 2013 and 2016. In addition, we will try to reveal the influencing factors in both private and public operational bodies. To this end, we analyze the care service indexes used in 2013 and 2016 in an attempt to establish the factors that affect user satisfaction with care services.

## 2. Materials and Methods

### 2.1. Framework for Research

The quality of care services in community care includes both objective and subjective factors. In research that emphasizes subjective factors, there is a viewpoint that the provided service is the user’s perception, and the aim is to induce the user’s satisfaction and confidence [28,29]. On the other hand, the quality of care services includes objective factors, such as access types to public services, visit frequency, and so forth [30]. There is a viewpoint as well that it encompasses all environmental factors, including systems and support organizations; structural factors, such as the operation and manpower of the providers; the service delivery process; the performance from the users’ perspective; etc. [31]. Accordingly, this study embraces both objective and subjective factors of community care services, and selected organizational operation, human management, service area, and evaluation as the influencing factors.

In South Korea, the quality evaluation of community care services is conducted every three years by the Social Security Information Service on the basis of these precedent studies for all providers. This study collected objective data used by the Social Security Information Service and used common variables as variables to measure service quality. In addition, the reason we comprehensively analyzed maternal newborn services, housekeeping and nursing services, and elderly services—which have different characteristics in terms of services—is that there are many institutions providing all three services, depending on what the provider is and that the evaluation is performed, centered on the provider, by the Social Security Information Service, a public institution.

**Figure 1:**
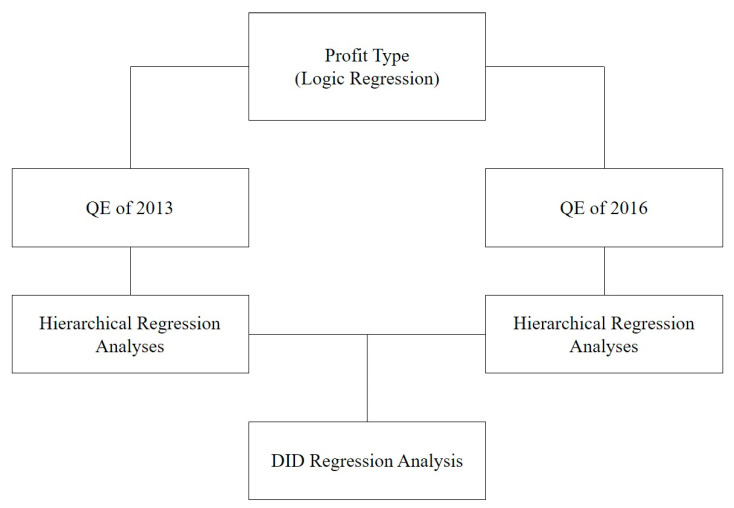
Research framework.

For the care services of social services, service QEs (Quality Evaluations) were conducted twice, in both 2013 and 2016, on the care for postpartum women and infants, house and health help, and older adult care. In this study, we will present directions for the successful implementation of community care projects in the future by comparing and analyzing the factors influencing care services in both years, looking at the change in the user satisfaction rates. The analytical framework to achieve this research goal is as follows (see Figure 1).

In particular, in that the country provides social services based on community care and the government evaluates the service quality by composing certain indicators, there is significance in comparing the influence factors on the service quality. Furthermore, in the 2 evaluations, DID regression analysis to analyze the influence factors on the score gap was performed to conduct an analysis on the change factors affecting the service quality.

First, this study acknowledges that leaning on just 423 institutions from a total population of 4331 may cause severe selection and survival biases, particularly self-selection biases in the second survey that will very likely cause endogeneity [32]. However, regarding the factors affecting whether or not a service is being conducted for profit, this study is analyzed using logit regression analysis. Second, the influencing factors in the care service evaluation indexes for 2013 and 2016 on user satisfaction are assayed separately via a hierarchical regression analysis. Third, a Difference in Differences (DID) regression analysis is conducted to identify the factors influencing the differences in the evaluation scores of care services in 2013 and 2016 and the user satisfaction with profit type and service type. In addition, the administrative dataset for this study only displays the presence or absence of revenue, type, and evaluation results, but does not present specific figures.

The hypotheses for testing in this study are as follows:

**Hypothesis** **1** **(H1).** *There will be a difference between the QE indexes of care services by profit type in 2013 and 2016.*

**Hypothesis** **2** **(H2).** *The care service evaluation indexes used in both 2013 and 2016 will affect user satisfaction*.

**Hypothesis** **3** **(H3).** *Differences in care service evaluation scores in 2013 and 2016 will have an effect on differences in their user satisfaction*.

### 2.2. Research Analyses

In this study, we used the following analyses to measure the internal consistency and validity of the QE (Quality Evaluation) of the care services. It is difficult to measure Heckman’s selection bias for the investigated subject of this study using a complete enumeration survey for providers on the government social service; the difference in the providing group by service type was not viewed, but the difference in evaluation scores according to the period of the same providers regardless of the service type was utilized. For the indicators used in this study, common variables were extracted among the variables used in the 2 service quality surveys, having non-standardized limited values.

First, a logit regression analysis was conducted to analyze the influencing factors for each profit type. Here, the profit type means whether it is a for-profit institution or not, and the logit analysis was used to analyze the influence factors on this through odds ratio, by using this profit type as a dependent variable. Second, in order to derive the QE (Quality Evaluation) indexes of the social service factors affecting user satisfaction, a regression analysis was performed separately for 2013 and 2016 to analyze the degree of influence. The hierarchical regression analysis was used here to verify the varying effect on the dependent variable as the independent variable changes, by classifying the influence factors of the independent variable on user satisfaction into four stages.

Third, a DID analysis was used to analyze the influence of the differences in index scores of providers, profit type, service type, etc., the common evaluation targets in 2013 and 2016, on the change in ratings for user satisfaction. DID regression analysis was used to analyze common variables that affect the change rate of user satisfaction. The specific formulas are as follows:

For the treatment of missing values in regression analysis, listwise deletion was used. In other words, the method was adopted whereby if even one value is missing, the entire record will be deleted. In this study, there were no missing values, because common providing organizations that participated in the evaluation in 2013 and 2016 were utilized as a sample.

$$\Delta CS=a+b\Delta X_{1}+c\Delta X_{2}+d\Delta X_{3}+\cdots P+S$$

CR=Client Satisfaction

$$X_{1}=Evaluation\;Index1,\;X_{2}=Evaluation\;Index2,\;X_{3}=Evaluation\;Index3$$

P=Profit Dummy, S=Services Dummy

### 2.3. Data Collection

For data collection, we utilized the manual for the QE (Quality Evaluation) of social services performed by the SSIS and secondary data quantified on the basis of the report analyzing the results. To this end, the commonly used indexes were selected on the basis of the evaluation indexes for care services in 2013 and 2016. In addition, to calibrate the differences in the added points between variables, as seen in the following, the scores were unified, or the weights were matched up (see Table 1).

Specifically, the Social Security Information Service, an affiliated organization of the Ministry of Health and Welfare, has conducted evaluations every three years in relation to the Social Service Quality Evaluation, which is considered to be the official administrative data in Korea. In 2013, 4 indices for institution operation, 5 indices for human management, 6 indices for service areas, and 1 index for on-site evaluation were used; in 2016, 3 indices for institution operation, 3 indices for human management, 6 indices for service areas, 2 indices for service performance, and 2 indices for on-site evaluation were utilized. However, in the Social Service Quality Evaluation in 2019, they were consolidated and reduced into 1 index for institution operation and 2 indices for human management. As the purpose of this study lies in comparing the indices for the Social Service Quality Evaluation, the evaluation indices for 2019 were excluded, because they differ significantly from those of the previous evaluation. In this study, the population is institutions that provide social services. Regarding the sampling process, the institutions for the 2013 evaluation, including 295 elderly care centers, 70 housekeeping and nursing service centers, and 89 maternal newborn service centers, were compared with the institutions for the 2016 evaluation, such as 428 elderly care centers, 96 housekeeping and nursing service centers, and 219 maternal newborn service centers; finally, 423 common providers were selected for the analysis object. Furthermore, for the analysis indices, 3 indices for institution operation, 2 indices for human management, 5 indices for service areas, and 1 index for on-site evaluation were also utilized, which were commonly used in the evaluations for 2013 and 2016.

Regarding the selection of analysis indicators, this study targeted the evaluation of social service institutions; it is not an analysis on the using gap (Johnson & Wolinsky, 1996) according to the group receiving the service, but relates to the service quality of the providers that deliver the social services offered by the country [33]. In addition, from the perspective of service consumers, personal variables were excluded: e.g., utilization rate, service delay, inappropriate use, etc. (Markle-Reid & Browne, 2001) [34].

On the other hand, as shown in the study of Plochg & Klazinga (2002), it is clarified that variables related to the role of the government providing services, such as process, organizational context, financial and policy context, etc. [35]—from the dimension of service user management—were also excluded from this study. In the case of South Korea, such social services based on community care are mainly led and performed by the government; organizations providing this should be regularly evaluated for the quality of their services. Therefore, this study was conducted under the basic premise that service satisfaction of users could be enhanced by improving the service quality of the provider.

## 3. Results

### 3.1. Differences in Influencing Factors by Profit Type

After using 1 for for-profit organizations and 2 for non-profit organizations in the service type in order to analyze the impact of the QE (Quality Evaluation) indexes of social services on profit type, a logit regression analysis was conducted. For the profit type, a total of 423 places of 88 for-profit organizations (20.8%) and 335 non-profit organizations (79.2%) were analyzed as an analysis subject. Based on the result of the analysis, the number of users, sales, accounting management, settlement disclosure, record management, contract termination, and tenure rates for the social service evaluation indexes of 2013 had a significance probability of less than 0.05. For the social service evaluation indexes, the number of users decreases by 0.986 for for-profit organizations; sales also increase by 1.000 for non-profit organizations. The reason non-profit organizations are more affected by sales than for-profit organizations is that in the case of Korea, most non-profit organizations are operated by fully depending on government subsidies. In terms of non-profit organizations, accounting management increases by 1 point, an increase by 3.333 times; as settlement disclosure increases by 1 point, profits increase by 2.917 times. For non-profit organizations, whenever record management and tenure rate increase by 1 point, they also increase by 4.040 and 2.142 times, respectively. By contrast, in the case of non-profit organizations, as the notice of contract terminations decreases by 1 point, each point represents a decrease by 0.185 times (See Table 2).

Second, in order to analyze the influence of profit type on care services in 2016, a logit regression analysis was conducted. The results showed a significance of less than 0.05 for number of users, tenure rate, and satisfaction rate. The tenure rate in the QE indexes of social services increases by 1.682 times for non-profit organizations whenever they increase by 1 point. In addition, whenever satisfaction increases by 1 point, satisfaction for non-profit organizations increases by 1.061 times. Furthermore, as the number of users increases by 1 point, the number of users of non-profit organizations decreases by 0.993 times (See Table 3).

In 2016, a logit analysis was performed on whether or not there was a for-profit organization as a dependent variable.

In conclusion, compared to those of 2013, the evaluation indexes of 2016 saw a reduction in the difference in influences between for-profit and non-profit organizations, which may suggest that they are fair indexes for both for-profit and non-profit organizations.

### 3.2. Influencing Factors on User Satisfaction

To analyze the influencing factors in the service QE indexes for user satisfaction in both 2013 and 2016, a hierarchical regression analysis was performed (see Table 4). The hierarchical regression analysis was conducted on each variable affecting user satisfaction, a dependent variable, based on the results of the Care Service Quality Evaluation in 2013 and 2016 conducted by the Social Security Information Service. The influence on the dependent variables was analyzed as the independent variables changed in four stages.

Above all, we analyzed the influencing factors in the care service evaluation indexes in 2013 for user satisfaction. In Model 1, profit type and service type were utilized as independent variables to analyze the influence on user satisfaction, but there were no significant factors.

In Model 2, the influencing factors on user satisfaction were analyzed using profit type, service type, sales, and number of users as independent variables, but there was no significant independent variable.

In Model 3, we analyzed the influencing factors of profit type, service type, sales, number of users, institutional operation area, human management area, service area, etc., on user satisfaction. The results showed that the longer the education time, the higher the tenure rate, the more clearly the contract termination was given, and the more thoroughly the document filing was performed, the higher the user satisfaction; these factors were statistically significant. However, the initial counseling and the counseling plan had a negative effect on user satisfaction.

In Model 4, by adding field evaluation indexes to profit type, service type, sales, number of users, institutional operation area, human management area, and service area, etc., we analyzed the influencing factors on user satisfaction. The results showed that the longer the education time, the higher the tenure rate, the clearer the contract termination was made, and the better the document filing, the higher the user satisfaction was.

The user satisfaction in 2013 tended to be high, at 0.9858 (standard deviation: 0.11853), and tolerance limits are represented as figures of 0.1 or higher, indicating no problem with multicollinearity. The Durbin Watson test was also 2.906, close to the standard of 2.0, which indicated that there was no autocorrelation. The change amount in the coefficient of determination (R^2^) did not show any significant changes going from step 1 to step 2 or from step 3 to step 4. However, when altering from step 2 to step 3, there was a statistically significant change: the coefficient of determination (R^2^) went from 0.07 to 0.95. In other words, it can be seen that the explanatory power is enhanced by the addition of institutional operation, human management, and service areas, which belong to the evaluation index areas, rather than service type, profit type, and performance.

Next, through a hierarchical regression analysis, we analyzed the magnitude of the influencing factors of the QE indexes for 2016 on user satisfaction (see Table 5). In Model 1, the influence on user satisfaction was analyzed using profit type and service type as independent variables; the user satisfaction increased as service type changed for postpartum women and infants, house and health help, and older adult care. Moreover, the difference was statistically significant.

In Model 2, we also analyzed the influencing factors on user satisfaction, using profit type, service type, sales, number of users, etc., as independent variables. The findings suggested that both profit and service types had a statistically significant influence. In other words, moving from for-profits to non-profits and from services for postpartum women and infants to older adult care services showed higher user satisfaction at a statistically significant level.

In Model 3, we analyzed the influencing factors for profit type, service type, sales, number of users, institutional operation area, human management area, service area, etc., on user satisfaction. The results suggested that moving to non-profits, moving from services for postpartum women and infants to older adult care services, and showing higher sales represented higher user satisfaction at a statistically significant level.

In Model 4, we analyzed the influencing factors on user satisfaction by adding field evaluation indexes to profit type, service type, sales, number of users, institutional operation area, human management area, service area, etc. The results showed that the closer to a non-profit, the closer to older adult care services and away from services for postpartum women and infants, the greater the number of users, and the higher the sales, the higher the user satisfaction, all at a statistically significant level. In particular, the higher the field evaluation scores, the higher the user satisfaction.

User satisfaction in 2016 was high, at 0.9196 (standard deviation: 0.04974), and the tolerance limit was over 0.1, showing no problem with multicollinearity. The Durbin Watson test also showed a result of 1.858, close to the standard figure of 2.0, which indicated that there was no autocorrelation. The change amount in the coefficient of determination (R^2^) did not show any significant changes while going from step 1 to step 2 and then to step 3. However, when changing from step 3 to step 4, there was a statistically significant change, with the explanatory power of the coefficient of determination (R^2^) decreasing from 0.20 to 0.05. In other words, field evaluation was a factor in reducing explanatory power of user satisfaction.

### 3.3. DID Hierarchical Regression Analysis

The factors influencing the rate of change in user satisfaction in the evaluation indexes of 2013 and 2016 were investigated through a DID hierarchical regression analysis, using profit type and service type as independent variables.

To measure the change amount in the scores of the evaluation indexes in 2013 and 2016, we subtracted the 2013 evaluation scores from the 2016 evaluation scores and then divided the result by the 2013 evaluation scores. We then multiplied the final figure by 100 to create the ratio. The specific formulas are shown below.

$$\mathrm{Change}\;\mathrm{Rate}=\left(\frac{\mathrm{Evaluation}\;\mathrm{Scores}\;\mathrm{of}\;2016-\mathrm{Evaluation}\;\mathrm{Scores}\;\mathrm{of}\;2013}{\mathrm{Evaluation}\;\mathrm{Scores}\;\mathrm{of}\;2013}\right)*100$$

Using a hierarchical regression analysis, we analyzed the magnitude of the influence of the change rate in QE scores for care services in 2013 and 2016 on the change rate in user satisfaction (see Table 6). In the case of Model 1, we analyzed the influence on user satisfaction using profit and service types as independent variables, where service type increased when moving from postpartum women and infants to house and health help and older adult care; there was a statistically significant influence.

In Model 2, we also analyzed the influencing factors of profit type, service type, sales, number of users, institutional operation area, human management area, service area, etc., on user satisfaction. The results suggested that the clearer the contract termination, the higher the user satisfaction, at a statistically significant level.

In Model 3, we analyzed the influencing factors on user satisfaction by adding field evaluation indexes to profit type, service type, sales, number of users, institutional operation area, human management area, service area, etc. The results showed that the clearer the contract termination, the higher the user satisfaction, and the results of field evaluations had a negative effect on user satisfaction.

The change rate in user satisfaction for 2013 and 2016 was –7.2747 (standard deviation: 4.66197), indicating lower user satisfaction in 2016. The tolerance limit represented 0.1 or higher, suggesting no problem with multicollinearity. The Durbin Watson test was also 2.224, close to 2.0, which indicated no autocorrelation. The change amount in the coefficient of determination (R^2^) did not show any significant changes while moving from step 1 to 2. However, when moving from step 2 to 3, the explanatory power of the coefficient of determination (R^2^) increased from 0.55 to 0.89, suggesting that there was a statistically significant change. In other words, it was found that the field evaluation increased explanatory power for the influence on user satisfaction.

## 4. Discussion

In this study, we analyzed the internal consistency and validity of the QE system of Korea’s care services by comparing evaluation indexes for 2013 and 2016. It can be argued that care services constitute the most important aspect in the community care project that has been in operation since July 2019 [5,36]. To analyze the factors affecting these care services, the influencing factors on the two profit types were analyzed using a logit regression analysis. In addition, to examine the influencing factors on user satisfaction, a hierarchical regression analysis was conducted, and in order to analyze the influencing factors in the rate of change in the evaluation scores of 2013 and 2016 for user satisfaction, a DID analysis was implemented.

With regard to the three hypotheses tested, Hypothesis 1, that there is a difference in influencing factors by profit type, was confirmed. Sales, accounting management, settlement disclosure, tenure rate, record management, etc., had a greater influence for non-profit organizations; number of users, contract termination, etc., had a greater influence for for-profit organizations [37,38]. It may be that because of the relatively high treatment levels of the services involving manpower in non-profit organizations, considerable motivation is given to sales, accounting management, tenure rates, etc.

Second, Hypothesis 2, that the care service evaluation indexes of 2013 and 2016 would separately affect the user satisfaction, was confirmed [39]. According to the hierarchical regression analysis for 2013, education time, attire management, contract termination, document filing, etc., had an effect on user satisfaction. In terms of the results of the hierarchical regression analysis for 2016, the closer the service type was to house and health help or to older adult care from postpartum women and infants, and the higher the sales, the more they affected user satisfaction. In addition, the higher the field evaluation scores were, the better the user satisfaction was. Therefore, in order to increase user satisfaction, it is necessary to differentiate between evaluation indexes by service type, and the quality of service should be improved via the expansion of education time. In general, however, it is necessary to solve the problem of the evaluation weight for user satisfaction being too high, as well as the fact that the evaluation scores vary according to the composition of the evaluation team. In addition, adjustment needs to be made in recognition of the fact that as the evaluation score has been raised excessively, the discrimination capacity in the evaluation scores is low [40,41].

Third, Hypothesis 3, that the change rate in the care service evaluation scores for 2013 and 2016 would affect the change rate in user satisfaction, was accepted. In particular, in Model 3, where there was a significant change in the coefficient of determination, the contract termination had a positive effect on user satisfaction; however, the field evaluation had a negative effect on user satisfaction. In other words, the effect of the field evaluation lowered the change in user satisfaction. This implies that it takes service providers excessive time to prepare the documents for the field evaluation. Accordingly, problems are occurring; for instance, companies that do paperwork for the field evaluation on behalf of service providers are utilized, suggesting that the current field evaluation system needs to be improved.

## 5. Conclusions

Thus far, we have compared and analyzed the differences in influencing factors on care service user satisfaction and whether they vary for profit type, so that community care projects can be implemented successfully. On the basis of the results, several implications can be drawn for improving satisfaction with care services in the future.

First, since, for the private sector, pursuing profits, number of users, and contract termination have an effect on user satisfaction, the feedback should be provided on the results of care service evaluations and strict post-treatments are required. In particular, customized guidelines should be presented with regard to improving caregivers’ labor conditions and evaluation results, which influence the quality of care services.

Second, the evaluation indexes by service type should be diversified and differentiated. In this study, we found that services for postpartum women and infants had significantly lower evaluation scores than those for house and health help or older adult care. In the case of companies that provide services for postpartum women and infants among social-service care projects in Korea, if most of them are mainly petty compared to institutions related to caring for old adults or to house and health help, they do not have systematic education or training (interview results with the evaluator). Furthermore, since those who have higher-income levels are using the private for-profit postpartum care center, there are many cases that the company providing services for postpartum women and infants is petty—operated by receiving subsidies from the government. Therefore, care service providers should be permitted to select indexes for themselves and be assessed on the indexes matching their institutional characteristics by expanding evaluation indexes and areas for regional and individual units, rather than analyzing all care services with the same indexes.

Third, with regard to the operation of evaluation teams, they should be organized by experts appropriate for each type of service, quantitative evaluations should be minimized, and field evaluations should mainly be related to user satisfaction. Moreover, by reinforcing the consultation functions rather than the evaluation authority of evaluation teams, the quality levels of care services should be enhanced, and care services appropriate to local characteristics should be provided.

Although this study is a study that empirically analyzed the differences according to the influence factors on community care services and the difference according to the profit types, it has the limitations as follows. First, it failed to consider all the various variables for evaluating service quality. Second, the care service analysis target was maternal newborn services, housekeeping and nursing services, and elderly care services, and the characteristics of each service were different, we analyzed them in an integrated manner. In addition, the dataset provided by the Social Security Information Service has a limitation in that the resulting value of the evaluation indexes is not classified by service type. Third, there are also limitations as follows: in order to investigate the influencing factors through the comparison of social service evaluation indices, only the common indicators were used among the evaluation indices for 2013 and 2016; during the selection process for analysis objects, only 423 places that have participated twice in the evaluation were selected. Among the 4,331 total institutions as a population parameter, services for mental health, the disabled, child protection, etc., were excluded from the evaluation, and there were only 423 institutions that had been evaluated simultaneously in 2013 and 2016.

Therefore, it should also be followed by evaluation of the services for mental health, the disabled, child protection, etc., as well as for the elderly. Nevertheless, it could be judged that there was an effort to maintain objectivity by using the dataset analyzed for the provider, utilizing the evaluation indexes of community care services, that was used by the responsible public institution in South Korea. In addition, governmental support will be more necessary to improve the quality of care services during the pandemic era, such as with COVID-19.

## Figures and Tables

**Table 1:** Common indexes for care service QE (Quality Evaluation).

Evaluation Area	Evaluation Index	2013		Evaluation Index	2016		Final Index	Points
		Detailed Index	Points		Detailed Index	Points		
Institutional Operation	Operating system	Arranging operational regulations Project operation plan	1 1	Operating system	Institutional operating regulations Project operation plan	1 1	Operational regulations Operation plan	1 1
	Information management	Privacy guidelines and education Security maintenance of personal information file	1 1	Information management	Personal information protection management Personal information security management	1 1	Information protection Information security	1 1
	Accounting management	Income and expenditure entry by service Once-a-year settlement statement disclosure	1 1	Accounting management	Accounting management by service Settlement statement disclosure	1 1	Accounting management Settlement disclosure	1 1
Human Management	Manpower management	Official recruiting process Salary provided under the labor contract Meeting eligibility qualification standards	1 1 1	Maintenance of recruitment	Fairness of recruitment Compliance with labor contracts Compliance with registration criteria	1 1 1	Recruiting process Labor contract Standard compliance	1 1 1
	Education system	In-house training for offered manpower External training for offered manpower	2	Education system	Yearly education time for offered manpower	2	Education time	2
Service Area	Service environment	Attire management for offered manpower	1	Service environment	Attire management for offered manpower	1	Attire management	1
	Tenure rate	Calculation of tenure rate for offered manpower	3	-	Tenure rate for offered manpower (divisions of manpower)	3	Tenure rate	3
	Plan establishment	Service provision plan for each user Description of Service provision schedule	1 1	Plan establishment and Contract conclusion	Initial counseling and service provision plan Record management for service provision	1 1	Counseling plan Record management	1 1
	Implementation and monitoring	Service satisfaction survey	1	Service performance	User satisfaction survey	1	Satisfaction	1
	Service linkage and termination	Cooperation with related institutions in community Provision of information on service termination Storage of service provision documents	1 1 1	Service linkage and termination	Connection with community Notice of service contract termination Storage of service provision documents	1 1 1	Community Contract termination Document filing	1 1 1
Field Evaluation Team	Organization chief’s leadership	Sense of duty and quality improvement in institutional operation Faithful preparation and creation of evaluation materials Consistency of self-evaluation report and evaluation materials	6	Overall evaluation	Organization chief’s efforts to improve service quality Degree of evaluation preparation Level of evaluation materials	2 2 2	Field evaluation	6

**Table 2:** Common indexes for QE (Quality Evaluation) of social services in 2013 (for-profit) (*n =* 423).

Dependent Variable	Independent Variable		Exp(B)	*p*
Profit Type	Performance	Number of users	0.986	0.001 ***
		Sales	1.000	0.001 ***
	Institutional operation	Operational regulations	1.524	0.366
		Operation plan	0.419	0.173
		Information protection	1.748	0.196
		Information security	0.318	0.152
		Accounting management	3.332	0.011 *
		Settlement disclosure	2.917	0.013 *
	Human management	Recruiting process	1.084	0.857
		Labor contract	1.401	0.644
		Standard compliance	5.819	0.259
		Education time	0.756	0.589
	Service area	Attire management	1.106	0.839
		Tenure rate	2.142	0.001 ***
		Counseling plan	0.632	0.369
		Record management	4.040	0.017 *
		Community	1.102	0.875
		Contract termination	0.185	0.003 **
		Document filing	0.396	0.431
	Field evaluation		1.031	0.823
	Satisfaction		0.869	0.916

Note: * *p* < 0.05, ** *p* < 0.01, *** *p* < 0.001.

**Table 3:** Common indexes for QE (Quality Evaluation) of social services in 2016 (for-profit). (*n* = 423).

Dependent Variable	Independent Variable		Exp(B)	*p*
Profit Type	Performance	Number of users	0.993	0.001 ***
		Sales	1.000	0.259
	Institutional operation	Operational regulations	0.801	0.811
		Operation plan	1.461	0.603
		Information protection	1.861	0.310
		Information security	1.002	0.999
		Accounting management	1.120	0.920
		Settlement disclosure	1.997	0.169
	Human management	Recruiting process	1.232	0.650
		Labor contract	1.991	0.258
		Standard compliance	0.985	0.987
		Education time	1.476	0.231
	Service area	Attire management	0.210	0.174
		Tenure rate	1.682	0.018 *
		Counseling contract	1.404	0.640
		Record management	0.311	0.425
		Community	1.183	0.757
		Contract termination	0.357	0.118
		Document filing	0.479	0.555
	Field evaluation		0.996	0.979
	Satisfaction		1.061	0.002 **

Note: * *p* < 0.05, ** *p* < 0.01, *** *p* < 0.001.

**Table 4:** Regression analysis for 2013. (*n* = 423).

Independent Variable	Model 1			Model 2			Model 3			Model 4		
	SE	β	t Value	SE	β	t Value	SE	β	t Value	SE	β	t Value
(Constant)	0.029		33.815	0.038		26.177	0.068		12.751	0.069		12.398
Profit type	0.018	−0.010	−0.167	0.018	−0.011	−0.185	0.019	0.040	0.623	0.019	0.037	0.579
Service type	0.018	−0.004	−0.058	0.023	−0.034	−0.438	0.026	−0.118	−1.339	0.027	−0.098	−1.095
Sales				0.000	0.134	1.389	0.000	0.115	1.213	0.000	0.111	1.165
Number of users				0.000	−0.074	−0.680	0.000	−0.075	−0.695	0.000	−0.072	−0.669
Operational regulations							0.018	0.021	0.405	0.018	0.032	0.597
Operation plan							0.024	0.067	1.172	0.024	0.071	1.255
Information protection							0.016	−0.056	−0.988	0.017	−0.044	−0.767
Information security							0.028	−0.067	−1.275	0.028	−0.074	−1.406
Accounting management							0.021	−0.062	−1.177	0.021	−0.061	−1.151
Settlement disclosure							0.018	−0.025	−0.428	0.018	−0.013	−0.219
Recruiting process							0.017	−0.090	−1.608	0.017	−0.078	−1.377
Labor contract							0.029	−0.029	−0.529	0.029	−0.034	−0.605
Standard compliance							0.054	−0.005	−0.101	0.054	0.001	0.010
Education time							0.019	0.115	1.996 *	0.019	0.117	2.031 *
Attire management							0.018	0.063	1.114	0.018	0.079	1.377
Tenure rate							0.008	0.096	1.808 **	0.008	0.104	1.946 **
Counseling plan							0.019	−0.088	−1.660 **	0.019	−0.085	−1.615
Record management							0.024	0.042	0.701	0.025	0.049	0.816
Community							0.025	−0.086	−1.435	0.025	−0.078	−1.308
Contract termination							0.021	0.166	2.749 *	0.021	0.170	2.827 *
Document filing							0.030	0.177	3.648 *	0.031	0.190	3.856 *
Field evaluation										0.005	−0.095	−1.423
Statistics	R^2^ = 0.000, F = 0.034			R^2^ = 0.007, F = 0.761			R^2^ = 0.092 *, F = 2.100 *			R^2^ = 0.005, F = 2.101 *		

Note: * *p* < 0.05, ** *p* < 0.01. Here, the value of R^2^ does not mean absolute R^2^, but the variance of R^2^ for each model phase; it was verified whether the variance had a significant change based on the F value.

**Table 5:** Regression analysis for 2016. (*n* = 423).

Independent Variable	Model 1			Model 2			Model 3			Model 4		
	SE	β	t Value	SE	β	t Value	SE	β	t Value	SE	β	t Value
(Constant)	0.011		74.700	0.017		47.824	0.034		24.527	0.035		24.460
Profit type	0.007	0.080	1.484	0.007	0.090	1.661 **	0.007	0.097	1.746 **	0.007	0.100	1.799 **
Service type	0.007	0.432	8.052 *	0.010	0.364	4.514 *	0.011	0.316	3.567 *	0.011	0.295	3.295 *
Number of users				0.000	−0.096	−1.071	0.000	−0.137	−1.472	0.000	−0.154	−1.657 **
Sales				0.000	0.106	1.586	0.000	0.122	1.799 **	0.000	0.126	1.852 **
Operational regulations							0.013	0.029	0.506	0.013	0.021	0.364
Operation plan							0.011	−0.014	−0.293	0.011	−0.017	−0.347
Information protection							0.010	0.021	0.432	0.010	0.017	0.337
Information security							0.025	−0.017	−0.311	0.025	−0.026	−0.496
Accounting management							0.016	−0.056	−1.164	0.016	−0.066	−1.362
Settlement disclosure							0.009	0.069	1.331	0.009	0.062	1.199
Recruiting process							0.007	0.037	0.789	0.007	0.038	0.821
Standard compliance							0.014	−0.015	−0.335	0.014	−0.019	−0.414
Education time							0.005	−0.045	−0.918	0.005	−0.057	−1.145
Tenure rate							0.004	−0.015	−0.334	0.004	−0.023	−0.522
Contract termination							0.009	0.074	1.491	0.009	0.073	1.459
Labor contract							0.010	0.035	0.738	0.010	0.035	0.733
Attire management							0.014	−0.011	−0.200	0.014	−0.017	−0.292
Counseling contract							0.011	−0.043	−0.843	0.011	−0.061	−1.177
Record management							0.014	−0.034	−0.750	0.014	−0.040	−0.867
Community							0.008	−0.047	−0.960	0.008	−0.063	−1.253
Document filing							0.019	0.018	0.383	0.019	0.019	0.395
Field evaluation										0.002	0.097	1.726 **
Statistics	R^2^ = 0.235, F = 64.454 *			R^2^ = 0.005, F = 32.911*			R^2^ = 0.020, F = 6.694 *			R^2^ = 0.005 **, F = 6.557 *		

Note: * *p* < 0.05, ** *p* < 0.01.

**Table 6:** Hierarchical regression analysis (DID). (*n* = 423).

Independent Variable	Model 1			Model 2			Model 3		
	SE	β	t Value	SE	β	t Value	SE	β	t Value
(Constant)	2.659		−5.551	3.479		−3.925	3.364		−3.322
Service type	1.509	0.186	2.255 *	1.953	0.156	1.464	1.870	0.111	1.085
Profit type	1.191	0.041	0.497	1.329	0.031	0.340	1.267	0.014	0.161
Number of users				0.015	−0.028	−0.210	0.014	−0.053	−0.423
Sales				0.006	0.009	0.078	0.006	0.018	0.159
Operation plan				0.028	−0.042	−0.486	0.027	−0.026	−0.314
Operational regulations				0.028	0.015	0.147	0.026	0.031	0.312
Information protection				0.017	0.081	0.915	0.016	0.108	1.273
Information security				0.040	0.054	0.610	0.038	0.036	0.433
Accounting management				0.037	−0.050	−0.526	0.035	−0.059	−0.657
Settlement disclosure				0.021	0.020	0.226	0.021	0.058	0.690
Recruiting process				0.016	0.068	0.765	0.015	0.105	1.224
Labor contract				0.022	−0.019	−0.232	0.021	−0.019	−0.253
Standard compliance				0.025	0.005	0.062	0.023	0.015	0.178
Education time				0.015	−0.028	−0.341	0.014	−0.017	−0.226
Attire management				0.032	−0.029	−0.281	0.030	0.006	0.065
Tenure rate				0.004	0.057	0.671	0.004	0.066	0.813
Counseling plan				0.037	−0.060	−0.733	0.035	−0.067	−0.857
Record management				0.030	0.028	0.332	0.029	0.006	0.071
Community				0.016	−0.098	−1.181	0.015	−0.090	−1.141
Contract termination				0.016	0.165	2.001 *	0.015	0.133	1.681 **
Document filing				0.079	−0.014	−0.158	0.075	−0.014	−0.169
Field evaluation							0.007	−0.318	−4.241 *
Statistics	R^2^ = 0.044, F = 4.213 *			R^2^ = 0.055, F = 0.865			R^2^ = 0.089 *, F = 1.728 *		

Note: * *p* < 0.05, ** *p* < 0.01.
